# *E pluribus unum*— Understanding Our Diversity and Disparities in Lp(a) Screening∗

**DOI:** 10.1016/j.jacadv.2024.100939

**Published:** 2024-05-01

**Authors:** Jaideep Patel, Harpreet S. Bhatia, Jared Alexander Spitz

**Affiliations:** a Johns Hopkins Hospital, Baltimore, Maryland, USA; b Ciccarone Center for the Prevention of Cardiovascular Disease, Johns Hopkins University School of Medicine, Baltimore, Maryland; c Division of Cardiovascular Medicine, University of California-San Diego, La Jolla, California, USA; d Inova Schar Heart and Vascular, Fairfax, Virginia, USA

**Keywords:** atherosclerosis, ethnic, lipid, Lp(a), prevention, risk

**Figure:**
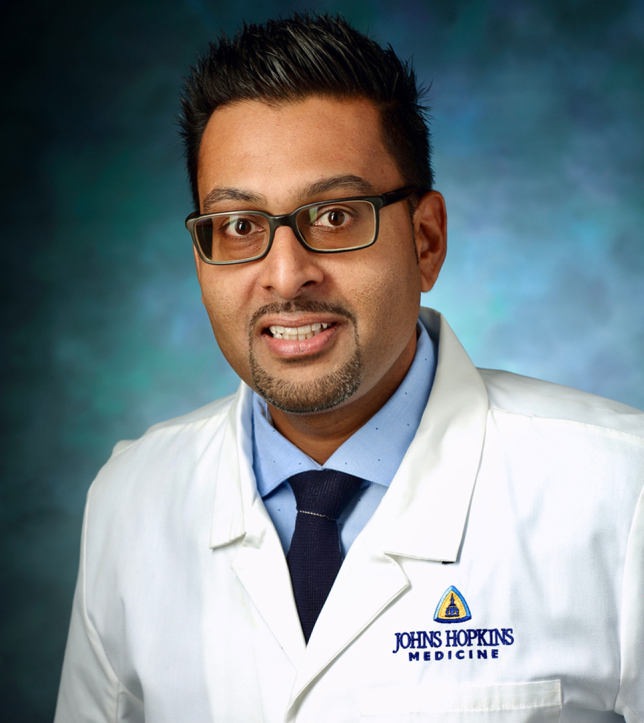

Lipoprotein(a) [Lp(a)] is a causal, genetic risk factor for atherosclerotic cardiovascular disease (ASCVD). However, it remains under-recognized as a risk factor and is infrequently screened for in real-world practice (Table 1). Rates of Lp(a) testing have ranged from 0.1% to 1% in retrospective studies to as high as 13.9% in the Lp(a)HERITAGE study.^3^, ^4^, ^5^, ^6^, ^7^ Commonly used cut points are Lp(a) levels >30 mg/dL or >50 mg/dL,^8^ which have been derived mainly from Caucasian populations. Lp(a) values vary by race and ethnicity, with the highest values seen in Black and South Asian adults. Limited data are available pertaining to differences in Lp(a) testing by race and ethnicity, which may serve as a potential barrier to Lp(a) screening overall.

**Table 1:** Studies of Prevalence of Lp(a) Testing

First Author/Year	Number of Participants	Years	Population	Overall Rate of Lp(a) Testing	Other Key Findings
Kelsey et al/2021^1^	--	2014-2019	Duke University Healthcare System	1,926	48.5% of those with Lp(a) testing had prior ASCVD
Nissen et al 2022^2^		2019-2021	Lp(a)HERITAGE study	48,135 (13.9%)	Enriched with patients with prior ASCVD being evaluated for enrollment in Lp(a) clinical trials.
Zafrir et al/2022^3^	∼4.6 million	2015-2021	Israel	4,539 (0.1%)	
Stürzebecher et al/2023^4^	4,672,811	2015-2018	Germany	45,095 (0.97%)	Testing more frequent in those with ASCVD in prior year
Kelsey et al/2023^5^	--	2015-2019	Eleven U.S. health systems	20,551 (0.1%)	Compared to those with LDL testing, the Lp(a) cohort more frequently had prevalent ASCVD and multiple prior CV events
Hu et al/2023^6^	330,964 secondary prevention; 1,314,838 primary prevention	2007-2020	Longitudinal U.S. Claims database	2,154 (0.7%) secondary prevention, 7,179 (0.5%) primary prevention	
Bhatia et al/2023^7^	5,553,654	2012-2021	6 University of California medical centers	18,972 (0.3%)	<4% in those with personal history of ASCVD 3.3% in those with family history of CVD
Dudum et al/2024^8^ (current study)	1,484,410	2010-2021	Sutter Health/Stanford University	13,689 (1.0%)	Asian and White groups, those with PPO insurance more likely to receive testing; women, Black or Hispanic individuals less likely. Black individuals tested more likely to have clinical ASCVD, while Asian Indian individuals were more likely to be at low 10-year risk. Elevated levels present to a similar degree across ASCVD risk spectrum when stratified by race/ethnicity

ASCVD = atherosclerotic cardiovascular disease; CVD = cardiovascular disease; Lp(a) = lipoprotein(a); PPO = Preferred Provider Organization.

Cardiovascular society guidelines are not consistent with recommendations for Lp(a) testing, ranging from no recommendation for universal screening in the U.S. Multi-Society Cholesterol Guidelines,^9^ to universal screening in European and Canadian society guidelines and statements.^10^, ^11^, ^12^ Mutual consensus lies in the use of Lp(a) as a risk-enhancing factor, however (Figure 1).^9^, ^10^, ^11^, ^12^, ^13^ In addition, there may be a lack of awareness of Lp(a) as a risk factor for ASCVD, concerns regarding insurance coverage, confusion related to differences between clinical assays and reporting practices, and a lack of understanding of how to manage individuals with elevated Lp(a) given the lack of available targeted therapies. However, elevated Lp(a) can impact risk assessment with management hinging on optimizing lifestyle and traditional factors, considerations for aspirin use, and intensification of lipid-lowering therapy.^12^^,^^14^

**Figure 1:**
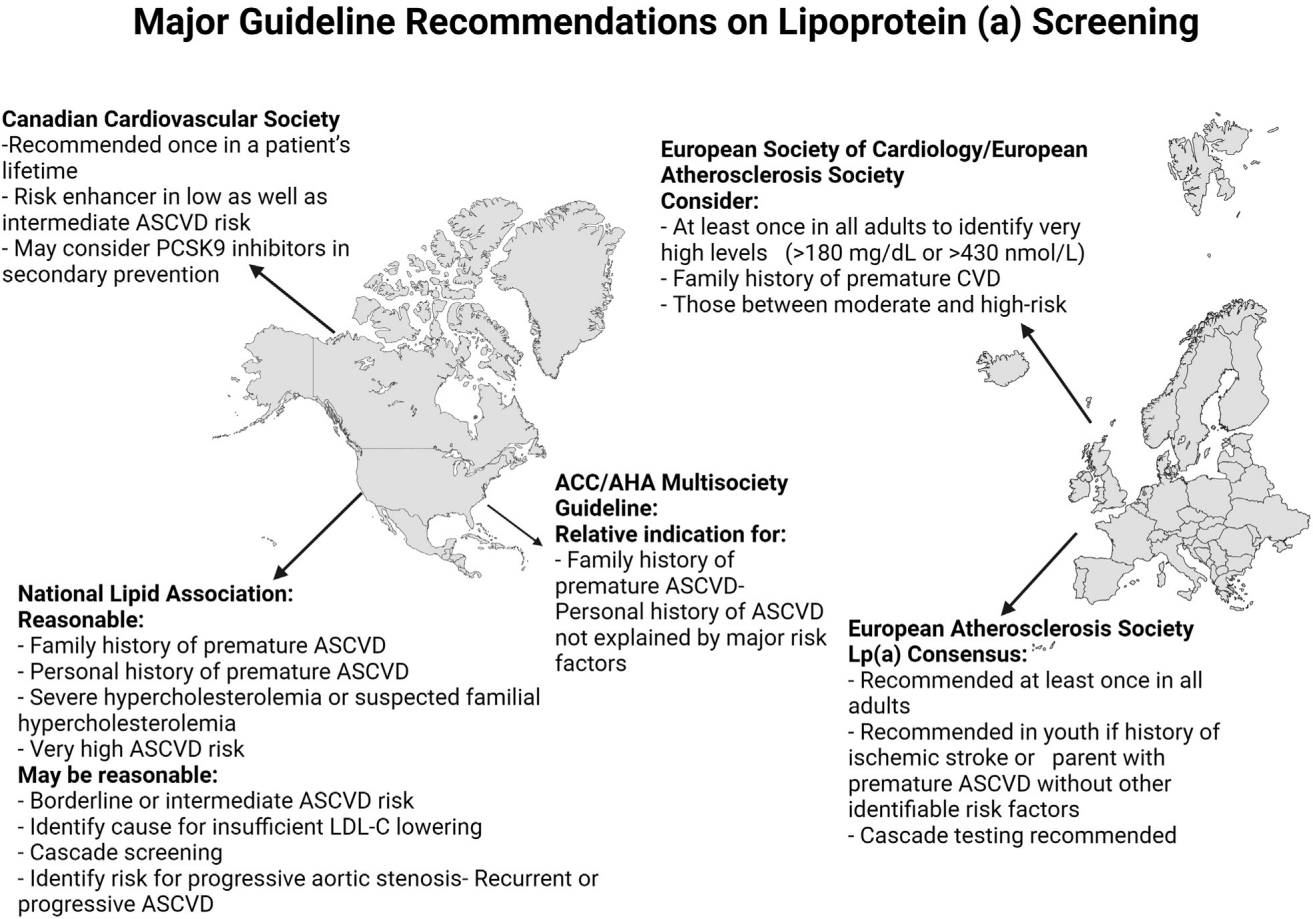
Society Recommendations for Lp(a) Testing ACC = American College of Cardiology; AHA = American Heart Association; ASCVD = atherosclerotic cardiovascular disease; CVD = cardiovascular disease; LP(a) = lipoprotein(a).

In this issue of *JACC: Advances*, Dudum et al^15^ describe racial and ethnic differences in Lp(a) testing in a real-word population. Utilizing a large health system, they evaluated 1,484,410 adult patients in California, USA, between 2010 and 2021. Impressively (but consistent with available data) only 1% underwent Lp(a) testing. Among non-Hispanic White, Black, Hispanic, and Chinese, Lp(a) testing was more common in women. Alternatively, men were most often screened among South Asian and other Asian groups. Black adults were more likely to have documented ASCVD, while Asian Indian individuals were more likely to be at low estimated risk and younger.^15^

Despite limitations in their data discussed below, Lp(a) distribution was similar when considering ASCVD risk categories, ASCVD risk factors, use of preventive therapies, and presence of established ASCVD. For those at low ASCVD risk, for whom statin therapy is not generally recommended, 61% and 53% had an Lp(a) 50 to 70 mg/dL and >70 mg/dL, respectively (cut point often used to identify “elevated” Lp(a)). Among Asian subgroups, Lp(a) 30 to 50 mg/dL and 50 to 70 mg/dL were most often seen in Asian Indian adults at 32% and 32%, respectively. The proportion of individuals with Lp(a) >50 mg/dL increased across ASCVD risk categories, ranging from 36% of individuals at low risk to 43% with a history of ASCVD.^8^

Novel to the literature was both insight into screening patterns and disaggregation of previously monolithic populations such as “Hispanic” and “Asian.” Overall, screening for Lp(a) remains low, with further disparities based on race/ethnicity. Black adults were most likely to have an ASCVD event, with testing completed to optimize secondary prevention efforts. Alternatively, South Asian adults were most often classified as low risk, with higher testing rates in the setting of improving primary prevention assessment; this is unsurprising considering the poor performance of the Pooled Cohort Equations in this group and a rise in awareness over the study period of higher rates of ASCVD events compared to other racial/ethnic groups.^16^ Additionally, they observed racial/ethnic variation in Lp(a) levels.

An overarching limitation of this study is selection bias, as testing was done based on clinical judgment, and therefore disaggregated median Lp(a) levels and distribution within subgroups need to be interpreted with caution. However, the overall trends are consistent with previously published data. Additionally, small numbers in certain race/ethnic groups could lead to misestimation of a median Lp(a).

The recently introduced PREVENT (Predicting Risk of cardiovascular disease EVENTs) risk calculator removed race as a risk factor for the development of ASCVD.^17^ While this is appropriate as race is a social construct, we cannot deny biological and non-biological factors such as social determinants of health and health care inequities that lead to differences in ASCVD rates.^18^ Variation in median Lp(a) levels by race may partially explain persistent differences in ASCVD risk once captured under race.^19^ Indeed, Lp(a) remains a causal risk factor for ASCVD on a per-particle basis, more so than non-Lp(a) apolipoprotein B100-containing particles.^20^^,^^21^ Given the noted prevalence of elevated Lp(a) in “low” and “borderline” risk groups and that the relative risk increase per increment of Lp(a) is the same across race/ethnicity, this supports universal Lp(a) testing as a way to further risk stratify individuals. In this setting, Lp(a) may serve as a reasonable indicator for genetic risk as to avoid the pitfall of overestimation or underestimation of risk (with the potential risk of worsening disparities) encountered with other risk estimators. In fact, a newly validated universal risk prediction model devoid of the race variable, leverages biomarkers such as Lp(a), and identifies higher risk persons who carry no known history of ASCVD.^22^ Validation of Lp(a) lowering and its effect on ASCVD events in upcoming studies will hopefully catalyze incorporation of LP(a) into risk prediction models.

Further studies regarding race/ethnicity specific cut points for Lp(a) are needed. Available evidence from another multiethnic cohort suggest ‘high’ Lp(a) threshold values of >50 mg/dL should be considered for White, Hispanic, and Chinese adults and >30 mg/dL for Black adults.^23^ There is also evidence that risk associated with Lp(a) rises linearly and specific cutoffs may not be warranted.^19^ The current study should spur future studies of racial and ethnic differences for other established associations of Lp(a) such as familial hypercholesterolemia,^24^ aortic stenosis,^25^ peripheral artery disease, and aortic aneurysms,^26^ for example.

As ongoing studies accrue about the relationship of Lp(a) with cardiovascular disorders and as targeted Lp(a) therapy becomes available, we anticipate increased Lp(a) testing congruent with international guidelines that advocate for universal screening. Considering racial and ethnic differences noted in the current body of work and disparities found in clinical care, we as a community need to be cognizant of this, working toward a better understanding of these differences and how to incorporate them into personalizing care. The authors take us a step in that direction.

## Funding support and author disclosures

Dr Bhatia was partially supported by 10.13039/100000002NIH 1K08HL166962; and has received consulting fees from Kaneka Medical and Novartis Pharmaceuticals. All other authors have reported that they have no relationships relevant to the contents of this paper to disclose.
